# Evidence for Health II: Overcoming barriers to using evidence in policy and practice

**DOI:** 10.1186/s12961-016-0086-3

**Published:** 2016-03-14

**Authors:** Anne Andermann, Tikki Pang, John N. Newton, Adrian Davis, Ulysses Panisset

**Affiliations:** Department of Family Medicine and Department of Epidemiology, Biostatistics and Occupational Health, Faculty of Medicine, McGill University, Montreal, Canada; Lee Kuan Yew School of Public Policy, National University of Singapore, Singapore, Singapore; Institute of Population Health, Faculty of Medical and Human Sciences, University of Manchester, Manchester, England; Public Health England, London, England; Department of Preventive and Social Medicine-Health Policy, Faculty of Medicine, Federal University of Minas Gerais, Belo Horizonte, Brazil; Evidence Informed Policy Network (EVIPNet) Steering Group, World Health Organization, Geneva, Switzerland

**Keywords:** Barriers, Decision-making, Evidence based medicine, Health policy, Public health, Research

## Abstract

Even the highest quality evidence will have little impact unless it is incorporated into decision-making for health. It is therefore critical to overcome the many barriers to using evidence in decision-making, including (1) missing the window of opportunity, (2) knowledge gaps and uncertainty, (3) controversy, irrelevant and conflicting evidence, as well as (4) vested interests and conflicts of interest. While this is certainly not a comprehensive list, it covers a number of main themes discussed in the knowledge translation literature on this topic, and better understanding these barriers can help readers of the evidence to be more savvy knowledge users and help researchers overcome challenges to getting their evidence into practice. Thus, the first step in being able to use research evidence for improving population health is ensuring that the evidence is available at the right time and in the right format and language so that knowledge users can take the evidence into consideration alongside a multitude of other factors that also influence decision-making. The sheer volume of scientific publications makes it difficult to find the evidence that can actually help inform decisions for health. Policymakers, especially in low- and middle-income countries, require context-specific evidence to ensure local relevance. Knowledge synthesis and dissemination of policy-relevant local evidence is important, but it is still not enough. There are times when the interpretation of the evidence leads to various controversies and disagreements, which act as barriers to the uptake of evidence. Research evidence can also be influenced and misused for various aims and agendas. It is therefore important to ensure that any new evidence comes from reliable sources and is interpreted in light of the overall body of scientific literature. It is not enough to simply produce evidence, nor even to synthesize and package evidence into a more user-friendly format. Particularly at the policy level, political savvy is also needed to ensure that vested interests do not undermine decisions that can impact the health of individuals and populations.

## Background

Billions of dollars are spent each year on producing research, but to what extent does all this research actually serve to improve health outcomes? Notwithstanding the value of creating knowledge for its own sake, it is difficult to justify spending limited resources on countless research studies, especially where there is potential for causing harm to research subjects (whether human or animal), if this does not contribute to a deeper understanding of the world we live in and how to make it a better place [1]. Even the highest quality evidence will have no impact unless it is incorporated into decision-making for health. Indeed, in recent years, there has been a push towards closing the ‘know-do’ gap – i.e. the gap between what we know works based on research evidence and what we actually do in practice [2]. There has also been an attempt to increase the likelihood of evidence being used to change policy and practice through co-production of knowledge between researchers and policymakers [3], as well as increasing the impact of research on improving health outcomes [4,5]. However, if our goal is to make more evidence-informed decisions that improve health, it is critical to be aware of the many barriers to using evidence in decision-making and to overcome these so that we can facilitate translating research evidence into improved health outcomes. The purpose of this article is therefore to highlight and better understand some of these barriers.

## Review

There exists a large and growing literature on the barriers to using evidence in decision-making for health. Some of the key themes that are often discussed include (1) missing the window of opportunity – often due to the relatively long time-frame required to generate new evidence and synthesize existing evidence and the relatively short time-frame available for making decisions, (2) knowledge gaps and uncertainty – especially the paucity of contextually-relevant evidence from local studies, (3) controversy, irrelevant and conflicting evidence – which act as smokescreens clouding the picture and making it difficult to decide how best to proceed, as well as (4) vested interests and conflicts of interest – which deliberately manipulate the evidence base to the detriment of the public’s health and wellbeing. These barriers often stem from the complexity inherent to producing knowledge, the disconnect between the worlds of researchers and policymakers, intentional subverting of the evidence for political or economic gain, or a combination of the above.

### Missing the window of opportunity

The first step in being able to use research evidence is ensuring that it is available at the right time and in the right format [6], so that knowledge users can take the evidence into consideration alongside a multitude of other factors that also influence decision-making.

The window of opportunity for incorporating evidence into decision-making can be very narrow, so unless the ‘work’ of having synthesized and packaged the evidence into a usable format has already been done beforehand, decisions are likely to be made without the aid of the large scientific literature that may be available. For instance, in the clinical setting, couples opting for prenatal screening for Down syndrome must make very difficult, morally-charged and potentially life-changing decisions in just a few weeks, and frontline health workers are making important treatment decisions on a daily basis within a 10–20 minute time window during each patient encounter. This explains why busy clinicians often turn to ‘pre-digested’ evidence-based resources such as *Up-to-Date*, *JAMA Evidence* and *MD Consult*, since these are quick and easy to use, as well as being clinically relevant, even if they come at a price. However, patients rarely have access to such tools, or even to information leaflets intended for patients which are not always distributed in practice, and therefore they must rely on what they can recall from the medical visit or else risk the information minefield which is the Internet. While ‘Googling’ for answers is certainly an option, there is a great deal of misleading information on the Internet. At the very least, it is important to use reputable websites and, ideally, sites that adhere to the HONcode principles, which is a code of ethics for quality health and medical information on the Internet [7].

In the policy world, the window of opportunity for decision-making can also be very constrained. Whether a controversy erupts in the media or an outbreak occurs, decisions must often be made under pressure. A good example of this is the policy response needed to deal with the H5N1 bird flu outbreak in Hong Kong in 1997. Margaret Chan, who was the Hong Kong Director of Health at the time, had to make a policy decision within 48 hours. She decided, based on her professional experience and convictions, to cull 1.5 million chickens in order to control the epidemic. While there are occasions when governments have the luxury of commissioning research from Health Technology Assessment agencies to aid decision-making, there are also occasions when the evidence is needed before the 5 pm press conference and there is simply no time to embark upon a systematic synthesis of the literature, even if one could call upon the experts to do so.

Thus, it is advisable to foresee common and/or important decisions that must be made – whether in a clinical setting or in a political context – and collect the best available evidence in advance to lay out the options and guide decision-makers through the pros and cons of these various options. Of course, decision-making is not straightforward, but rather a dynamic and non-linear process [8], and evidence is only one type of input that goes into this process (which also integrates many other considerations such as valuations, preferences, feasibility, cost, etc.). Nonetheless, evidence is more likely to be used when it is available during the window of opportunity, if it points to options which are actionable and if the existing resources and infrastructure can be used to make it happen, rather than requiring an influx of new resources [4]. For instance, one of the reasons for the success of the Thai universal health coverage policy, in addition to the solid evidence behind it, was that the proponents underlined that it could be done within the existing budget.

### Knowledge gaps and uncertainty

Another major barrier to using evidence for informing policy and practice is the lack of available evidence in specific areas or in specific contexts. This can be extremely problematic. Particularly as we move away from strictly clinical questions, such as the best pharmaceutical treatment for hypertension or the best diagnostic test for colon cancer, and attempt to address broader social questions, such as how to tackle gender-based violence and how to create supportive environments for health, we enter a realm where it is often difficult to be entirely evidence-based because the evidence has not been produced to the same extent. Since research requires funding, and a great deal of research is privately funded in part or in whole, it is not surprising that there is relatively little research on issues such as gender-based violence, child maltreatment or health inequities more broadly, since there is no pill or product that can be used to manage these health-related problems. Similarly, when dealing with specific under-served populations, such as immigrants or Indigenous groups, one may also run into the same problem that there is often relatively little research evidence on ‘what works’ to guide action in these specific populations, not to mention the glaring research gap with respect to low- and middle-income countries [9]. Even when there is solid evidence at an international level, policymakers, especially in resource-constrained settings, often lack the local evidence relevant to their particular context which would be important in helping them make more informed decisions for improving the health of their local populations given the particular circumstances and challenges that they face.

A lack of evidence does not mean that the interventions or strategies in question are not effective. It simply means that they have not been sufficiently investigated. The bottom line is that, in the absence of evidence, one is left with a great deal of uncertainty under which decisions must nonetheless be made. Therefore, at the very least, it is necessary to gather together the best possible evidence and to at least make explicit the knowledge gaps. Indeed, there is no such thing as zero uncertainty and therefore it is simply a matter of degree. Often pleas for more research, while helpful, will generally not provide the additional evidence required within the timeline necessary to make decisions. Nonetheless, in the meantime, decision-makers must resort to examining best practices or examples of what has worked in different contexts to make the best possible decisions in situations where there is a lack of guidance from the existing body of research evidence.

### Controversy, irrelevant and conflicting evidence

**Fig. 1:**
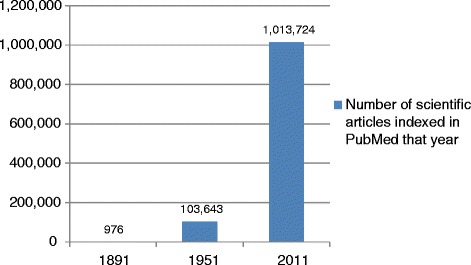
**The exponential increase in research evidence over the last century.** Data based on articles indexed PubMed (http://www.ncbi.nlm.nih.gov/pubmed/)

It is a strange paradox that, although we suffer from important knowledge gaps regarding how to improve population health, the scientific literature is filled with a plethora of ‘irrelevant evidence’ that is ‘nice to know’ but not really what we ‘need to know’ to improve health. Research topics chosen by researchers tend to be of academic interest, and only loosely driven by the information needs of patients or decision-makers, if at all. It is therefore not surprising that the evidence produced then tends to have little relevance or practical value for patient choices or policymaking. According to the Agency for Healthcare Research and Quality [10], before implementing a new intervention, policymakers want to know (1) can it work? (2) will it work here? and (3) is it worth it? Yet, one must spend time going through a great deal of irrelevant evidence to find patient-informed, policy-friendly data that helps to answer these questions. As the number of research publications continues to grow exponentially (Fig. 1), it is becoming virtually impossible to keep abreast of the scientific literature in any given field [11].

Beyond the lack of relevant evidence and the ‘noise’ created by irrelevant evidence, another challenge for decision-making is disagreement about what the evidence says. While generally evidence-based recommendations are quite similar across jurisdictions and contexts [12], there are occasions when different groups make recommendations based on the same international body of evidence which are clearly at odds with the recommendations made elsewhere. This can lead to significant confusion and contentious debate, which undermines confidence in the scientific process, and is a major barrier to the uptake and use of evidence.

The mammography controversy is a case in point [13]. When the Nordic Cochrane Centre published a systematic review in 2001 stating that “*currently available reliable evidence does not show a survival benefit of mass screening for breast cancer*” it caused a great outcry [14]. There were many articles written condemning anyone who dared to question the clinical utility of breast screening as being part of an “*active anti-screening campaign… based on erroneous interpretation of data from cancer registries and peer-reviewed articles*” [15]. Indeed, unlike the other reviews of breast cancer screening, the Nordic review excluded certain studies for methodological reasons and included others, thereby arriving at a different conclusion, which is certainly legitimate. However, the interpretation of the authors went a little too far to counteract the then prevailing school of thought on the benefits of screening. A few years later the Nordic Cochrane Centre updated their analysis and revised their conclusions to be more nuanced. The most recent update of the review in 2011 states that “*screening is likely to reduce breast cancer mortality*” – but, they qualify this statement with an explanation [16]. The authors conclude that the mortality reduction attributable to screening is much lower than previously suggested by reviews of the mammography literature (i.e. 15% versus 30%). Further, overdiagnosis and overtreatment are much more common than is generally acknowledged. Thus, without rejecting breast screening altogether, the Nordic Cochrane Centre provides the data for patients and policymakers to better weight the benefits and harms. If people invited for screening only knew that, out of 2,000 women screened for 10 years, only one will avoid dying from breast cancer, whereas 10 will receive unnecessary treatment, and over 200 will experience a false alarm requiring repeat testing and invasive diagnostic procedures, they would be better equipped to judge for themselves the merits of participating in the screening program [17]. Indeed, if they choose to participate, they may even find this information reassuring by knowing in advance that false alarms are common and that only a small proportion of women who are recalled for additional testing actually have cancer.

Even though we all share the same international body of research evidence, there is a great deal of subjectivity in the analysis of that evidence [18]. There are times when subtly different approaches to interpretation leads to various controversies and disagreements, which act as barriers to the uptake of evidence, whereas people should be presented the evidence with all its nuances and complexity, so that they can better choose for themselves. Since the body of evidence is not static, and the knowledge base is constantly being revised and refined, these controversies prove to be counter-productive and claims that a single study can overturn decades of research are misguided. Instead, the focus should be on the quality and rigour of examining large bodies of evidence produced over time and producing nuanced information that is helpful for decision-makers [19]. However, of even greater concern is when the evidence base is deliberately manipulated and misused with the intention of promoting certain vested interests, as discussed in the following section.

### Vested interests and conflicts of interest

The degree to which vested interests have infiltrated the international body of evidence is not to be underestimated. Often the way in which these interests play out in the literature can be quite subtle. However, astute readers of the literature should consider the underlying motives behind research publications, and what the authors stand to gain or lose. In areas such as tobacco smoking, the introduction of new pharmaceuticals and the manufacture and export of asbestos, the research evidence can make or break an industry, with millions of dollars as well as thousands of jobs at stake. Therefore, there is a great deal to lose and tremendous incentive for these mega-industries to prevent the diffusion of studies which go against their commercial interests or even actively infuse the larger body of evidence with conflicting studies to raise some doubt as to whether their product is indeed harmful – sufficient doubt to allow the money-making enterprise to continue reaping profits, even if only for a few more years. This sounds very sinister and hard to believe, yet, there are regular accounts of just this.

Indeed, a great deal has been written over the years about how the tobacco industry has attempted in various ways to call into question the link between smoking and cancer by producing their own evidence – often flawed or misinterpreted to their own ends [20]. The industry is known to have co-opted a large number of “*venal or naive scientists*” [21] and even commissioned consultants to write articles that call into question the link between second hand smoke (SHS) and sudden infant death syndrome (SIDS). According to internal memos made public during legal action against the tobacco industry, there is proof that tobacco company executives “*successfully encouraged one author to change his original conclusion that SHS is an independent risk factor for SIDS to state that the role of SHS is ‘less well established’…*” [22]. The author’s disclosure of industry funding did not reveal the full extent of the company’s involvement in shaping the content of the article. Yet another example of how accepting industry funds can disrupt the integrity of the scientific process.

However, the tobacco industry is not the only culprit. A great deal has also been written in the medical literature about the pharmaceutical industry introducing bias which could have a favourable impact on medication sales and ultimately increase profits for shareholders. For instance, through “*multiple publication of positive trials and non-publication of negative trials, reinterpreting data submitted to regulatory agencies, discordance between results and conclusions,* [and] *conflict-of-interest leading to more positive conclusions*” [23].

Additionally, cash-strapped universities are increasingly looking for alternative routes to fund-raising and attempt to capitalize on the production of intellectual property. Indeed, some universities have even developed venture capital teams to support the roll-out and marketing of scientific discoveries and technological innovations developed on their campuses. Yet, a commitment to open scientific inquiry and the pursuit of financial gains are two goals that can be very difficult to reconcile, often requiring a number of safeguards to be in place [24]. Even beyond academia, governments are also implicated. In the interest of keeping the economy running and creating more jobs, governments are often eager supporters of new industrial sectors. For instance, the Canadian government is pouring money into genome research [25], even though benefits to human health are still unproven [26]. At times, these business ventures are even shown to have serious negative consequences for human health and well-being [27]. Nevertheless, any evidence of such links must be quashed to avoid interfering with economic gains and even with re-election. Therefore, in certain situations, governments have been accused of obfuscating the truth [28]. For instance, according to an editorial in the *Canadian Medical Association Journal* [29], “*Canada is the only Western democracy to have consistently opposed international efforts to regulate the global trade in asbestos. And the government of Canada has done so with shameful political manipulation of science*”. However, Canada is not alone when it comes to benefitting from ‘overlooking’ the evidence when it is inconvenient. Many countries, including Mexico, Indonesia and China, in spite of having ratified the Framework Convention on Tobacco Control, continue to receive so much income from the sale of these products through ‘sin taxes’ and other channels that they have difficulty implementing measures intended to reduce the use of tobacco products within their borders with the aim of improving the health of their own people [30].

Even the ways in which health systems are organized and run are not immune to the misappropriation of evidence. Universal access to publicly-funded healthcare is an important determinant of health [31] and “*a privatized, ‘American-style’ health financing and provision system is neither a feasible nor desirable model for developing countries*” [32]. Nonetheless, according to Whitehead et al. [33], “*In the past two decades, powerful international trends in market-oriented health-sector reforms have been sweeping around the world… advocated by agencies such as the World Bank to promote privatisation*”. Thus, the authors call for an evidence-based approach to health sector reform, rather than promoting mass privatization of healthcare services and charging poor people user fees, which have been shown time and again to have negative impacts on health [34,35]. Interestingly, under new leadership, even the World Bank has done an abrupt turn in support of universal health coverage, which “*suggests that an evidence-based approach to policy may finally be prevailing over an ideologically driven approach*” [36]. Thus, there is hope that even the most powerful vested interests can be overcome.

## Conclusions

Readers of the scientific literature should be aware of the ways in which the research evidence can be influenced and misused for various aims and agendas. That is not to say that we should throw out the whole ‘evidence-based’ enterprise, but simply that a healthy dose of scepticism and critical thinking is advisable. It is not enough to simply produce evidence, nor even to synthesize and package evidence into a more user-friendly format. New evidence needs to be critically appraised and considered in light of the larger body of existing scientific literature, both local and international. Particularly at the policy level, political savvy is also needed to ensure that vested interests do not undermine decisions that can impact the health of individuals and populations. Whether it is tobacco companies trying to flood the literature with contradicting evidence or pharmaceutical companies hiding research demonstrating that their products have no effect or lead to harm, these conflicts of interest can lead us to make erroneous conclusions and misinformed decisions. Ultimately, the public pays the price, whether through poor health or misspent money.

In response to these challenges, a growing number of organisations, agencies, task forces and even global networks have been developed to synthesize, appraise and disseminate research evidence with the goal of improving health and reducing inequities. These include the Cochrane and Campbell Collaborations [37], Health Technology Assessment Agencies [38], the Canadian Task Force on Preventive Health Care [39], and the US Preventive Services Task Force [40], all of which focus more on clinical-level guidance, as well as the Community Guide, which focuses more on population-level health interventions [41]. However, while finding, appraising and synthesising the evidence and ‘putting it out there’ in a clinical practice guideline, on a website or in a database is important, it is often not sufficient to ensure that the evidence will be used to influence policy and practice. Increasingly, it is being recognised that evidence needs to be packaged in such a way so as to actively promote uptake, since passive diffusion measures are much less likely to have an impact [42].

Many different models are being developed to increase the uptake and use of evidence in practice [43]. These often involve some form of evidence summaries or decision-support tools. For instance, the EVIPNet Portal includes a repertory of EVIPNet Policy Briefs which synthesise the research evidence and offer evidence-informed and contextualised policy options in a user-friendly format to support well-informed policy decisions [44]. Public Health England’s *Longer Lives*/*Healthier Lives* website is another example that provides statistical data tools that allow people to see how their local area compares to the rest of the country in terms of specific health indicators and provides a route to existing evidence summaries produced by the UK’s National Institute for Health and Clinical Excellence [45]. Indeed, such policy briefs, which are free from technical jargon and highlight key messages in a brief executive summary, dramatically increase the likelihood that policymakers will read, consider and apply the evidence where appropriate. The EVIPNet partners with multiple organisations to produce these policy-relevant evidence syntheses, including the Alliance for Health Policy and Systems Research, the Health Evidence Network, and Supporting Policy relevant Reviews and Trials. Similarly, the Cochrane Collaboration produces Cochrane Summaries’ to make their systematic reviews more readily accessible to a wider audience of knowledge users [46].

While it is important to be aware of the barriers and facilitators when using evidence in decision-making for health, at the end of the day, a decision must be made which takes into account the needs and concerns of the individual patients and local populations involved. In the next article in the series, a model for ensuring more participatory, transparent and evidence-informed decisions will be presented and discussed.

## References

[1] Andermann A (2013). Evidence for Health: From Patient Choice to Global Policy.

[2] Rycroft-Malone J (2013). From knowing to doing-from the academy to practice: Comment on “The many meanings of evidence: implications for the translational science agenda in healthcare.”. Int J Health Policy Manag.

[3] Lehmann U, Gilson L (2015). Action learning for health system governance: the reward and challenge of co-production. Health Policy Plan.

[4] Greenhalgh T, Fahy N (2015). Research impact in the community-based health sciences: an analysis of 162 case studies from the 2014 UK Research Excellence Framework. BMC Med.

[5] Redman S, Haynes A, Williamson A (2015). Research impact: neither quick nor easy. BMC Med.

[6] Muir Gray JA (2001). Evidence-based Health Care.

[7] Safe Use of Internet. Chene-Bourg: Health on the Net Foundation; 2011. http://www.hon.ch/HONcode/Patients/visitor_safeUse2.html. Accessed 11 February 2016.

[8] Nutbeam D, Harris E (2004). Theory in a Nutshell: A Practical Guide to Health Promotion Theories.

[9] Commission for Health Research on Development (1990). Health Research: Essential Link to Equity in Development.

[10] Brach C, Lenfestey N, Roussel A, Amoozegar J, Sorenson A (2008). RTI International. Will It Work Here? A Decisionmaker’s Guide to Adopting Innovations.

[11] Garba S, Ahmed A, Mai A, Makama G, Odigie V (2010). Proliferations of scientific medical journals: a burden or a blessing. Oman Med J.

[12] Mavriplis C, Thériault G (2006). The periodic health examination: a comparison of United States and Canadian recommendations (French). Can Fam Physician.

[13] Finkel M (2005). Understanding the Mammography Controversy: Science, Politics and Breast Cancer Screening.

[14] Olsen O, Gøtzsche PC (2001). Screening for breast cancer with mammography. Cochrane Database Syst Rev.

[15] Bock K, Borisch B, Cawson J, Damtjernhaug B, de Wolf C, Dean P (2011). Effect of population-based screening on breast cancer mortality. Lancet.

[16] Gøtzsche PC, Nielsen M (2011). Screening for breast cancer with mammography. Cochrane Database Syst Rev.

[17] Gotzsche P, Hartling O, Nielsen M, Broderson J, Jorgensen K (2009). Breast screening: the facts—or maybe not. BMJ.

[18] Subjectivity in data analysis. Lancet. 1991;337(8738):401–2. 1671431

[19] AGREE Collaboration (2003). Development and validation of an international appraisal instrument for assessing the quality of clinical practice guidelines: the AGREE project. Qual Saf Health Care.

[20] Tong EK, Glantz SA (2007). Tobacco industry efforts undermining evidence linking secondhand smoke with cardiovascular disease. Circulation.

[21] Chapman S, Shatenstein S (2001). The ethics of the cash register: taking tobacco research dollars. Tob Control.

[22] Tong EK, England L, Glantz SA (2005). Changing conclusions on secondhand smoke in a sudden infant death syndrome review funded by the tobacco industry. Pediatrics.

[23] Lexchin J (2012). Those who have the gold make the evidence: how the pharmaceutical industry biases the outcomes of clinical trials of medications. Sci Eng Ethics.

[24] Omobowale EB, Kuziw M, Naylor MT, Daar AS, Singer PA (2010). Addressing conflicts of interest in Public Private Partnerships. BMC Int Health Hum Rights.

[25] Harper Government Invests in Personalized Medicine [press release]. Genome Canada. 2012. http://www.genomecanada.ca/en/about/news.aspx?i=409. Accessed 11 February 2016.

[26] Wade N. A Decade Later, Genetic Map Yields Few New Cures. New York Times. 2010 (June 13). http://www.nytimes.com/2010/06/13/health/research/13genome.html. Accessed 11 February 2016.

[27] Nuffield Council on Bioethics (2010). Medical Profiling and Online Medicine: The Ethics of ‘Personalised Healthcare’ in a Consumer Age.

[28] Kazan-Allen L (2003). The asbestos war. Int J Occup Environ Health.

[29] Attaran A, Boyd D, Stanbrook M (2008). Asbestos mortality: a Canadian export. CMAJ.

[30] Madrazo-Lajous A, Guerrero-Alcántara A (2012). Undue tobacco industry interference in tobacco control policies in Mexico. Salud Publica Mex.

[31] Van Lerberghe W, Evans T, Rasanathan K, Mechbal A, Andermann A, Evans D (2008). World Health Report 2008 - Primary Health Care: Now More than Ever.

[32] Hozumi D, Frost L, Suraratdecha C, Pratt B, Sezgin Y, Reichenbach L (2008). The Role of the Private Sector in Health: A Landscape Analysis of Global Players’ Attitudes toward the Private Sector in Health Systems and Policy Levers That Influence These Attitudes.

[33] Whitehead M, Dahlgren G, Evans T (2001). Equity and health sector reforms: can low-income countries escape the medical poverty trap?. Lancet.

[34] Tipping G (2000). The social impact of user fees for health care on poor households: commissioned report to the Ministry of Health.

[35] Stuckler D, King L, McKee M (2009). Mass privatisation and the post-communist mortality crisis: a cross-national analysis. Lancet.

[36] Rowden R (2013). The ghosts of user fees past: exploring accountability for victims of a 30-year economic policy mistake. Health Hum Rights.

[37] The Cochrane Collaboration. http://www.cochrane.org. Accessed 11 February 2016.

[38] International Network of Agencies for Health Technology Assessment (INAHTA). http://www.inahta.net/. Accessed 11 February 2016.

[39] Canadian Task Force on the Periodic Health Examination (1994). Canadian Guide to Clinical Preventive Health Care.

[40] US Preventive Services Task Force (USPSTF) (2010). Guide to Clinical Preventive Services, 2010–2011.

[41] Community Preventive Services Task Force (2005). The Guide to Community Preventive Services: What Works to Promote Health.

[42] Lavis J, Davies H, Oxman A, Denis JL, Golden-Biddle K, Ferlie E (2005). Towards systematic reviews that inform healthcare management and policymaking. J Health Serv Res Policy.

[43] Waters E, Armstrong R, Swinburn B, Moore L, Dobbins M, Anderson L (2011). An exploratory cluster randomised controlled trial of knowledge translation strategies to support evidence-informed decision-making in local governments (The KT4LG study). BMC Public Health.

[44] EVIPNet Portal. Geneva: World Health Organization; 2012. http://www.evipnet.org/modules/dia/index.php?where=POLICYBRIEF. Accessed 11 February 2016.

[45] Longer Lives/Healthier Lives. London: Public Health England; 2014. http://healthierlives.phe.org.uk/topic/mortality. Accessed 11 February 2016.

[46] The Cochrane Collaboration (2012). Cochrane Summaries.

